# OSTIR: open source translation initiation rate prediction

**DOI:** 10.21105/joss.03362

**Published:** 2021-08-22

**Authors:** Cameron T. Roots, Alexandra Lukasiewicz, Jeffrey E. Barrick

**Affiliations:** 1 Department of Molecular Biosciences, Center for Systems and Synthetic Biology, The University of Texas at Austin; 2 McKetta Department of Chemical Engineering, The University of Texas at Austin

## Abstract

Translation of messenger RNAs into proteins by the ribosome is a fundamental step in gene expression. In bacteria, it is possible to accurately predict the rate of translation initiation from the sequence surrounding a gene’s start codon using thermodynamic models of RNA folding and ribosome binding. These predictions have applications in a range of fields, from systems biology studies that aim to understand and model bacterial physiology to synthetic biology studies that seek to reprogram bacterial cells. For example, metabolic engineers can design ribosome binding site (RBS) sequences to tune the expression of different enzymes in a pathway and thereby optimize the production of a desired chemical compound by cells. OSTIR (Open Source Translation Initiation Rates) is a Python package and command line tool for predicting translation initiation rates in bacteria.

## Statement of Need

Several software programs exist for predicting translation initiation rates in bacteria (Reis & Salis, 2020), but none of these alternatives is a fully open source solution. Though the RBS Calculator v1.0 (Salis et al., 2009, 2015; Salis, 2011) is open source (GPLv3), it is not maintained and is only functional when using the NUPACK software suite for RNA structure energy calculations (Zadeh et al., 2011). NUPACK is not open source and has licensing restrictions. Further updates to the RBS Calculator code beyond version 1.0 (Espah Borujeni et al., 2014; Reis & Salis, 2020) are proprietary, and this software can only be used to make predictions through a web server that requires user registration (De Novo DNA, 2020). Thus, the RBS Calculator cannot be freely used as part of a software pipeline or further improved by the open source community. Other software programs for predicting translation initiation rates have similar restrictions. For example, RBSDesigner is distributed only as an executable and uses another RNA folding software suite that requires a license (Na & Lee, 2010).

## Implementation

OSTIR is open source software (GPLv3) derived from the RBS Calculator v1.0 codebase (Salis et al., 2009, 2015; Salis, 2011). OSTIR was rewritten to use the open source ViennaRNA software suite (Lorenz et al., 2011) to perform the necessary RNA structure energy calculations so that its entire workflow is open source. OSTIR also features several improvements in usability and flexibility over the RBS Calculator v1.0 and related tools that include: (1) OSTIR and its ViennaRNA dependency can be easily installed through Bioconda (Grüning et al., 2018); (2) OSTIR supports multithreading to accelerate the analysis of large sequences and genomes; (3) OSTIR allows the user to specify the anti-Shine-Dalgarno sequence used for the ribosome so that predictions can be made for bacterial species other than *Escherichia coli*; (4) OSTIR supports multi-FASTA and CSV input files for batch processing.

**Figure 1:**
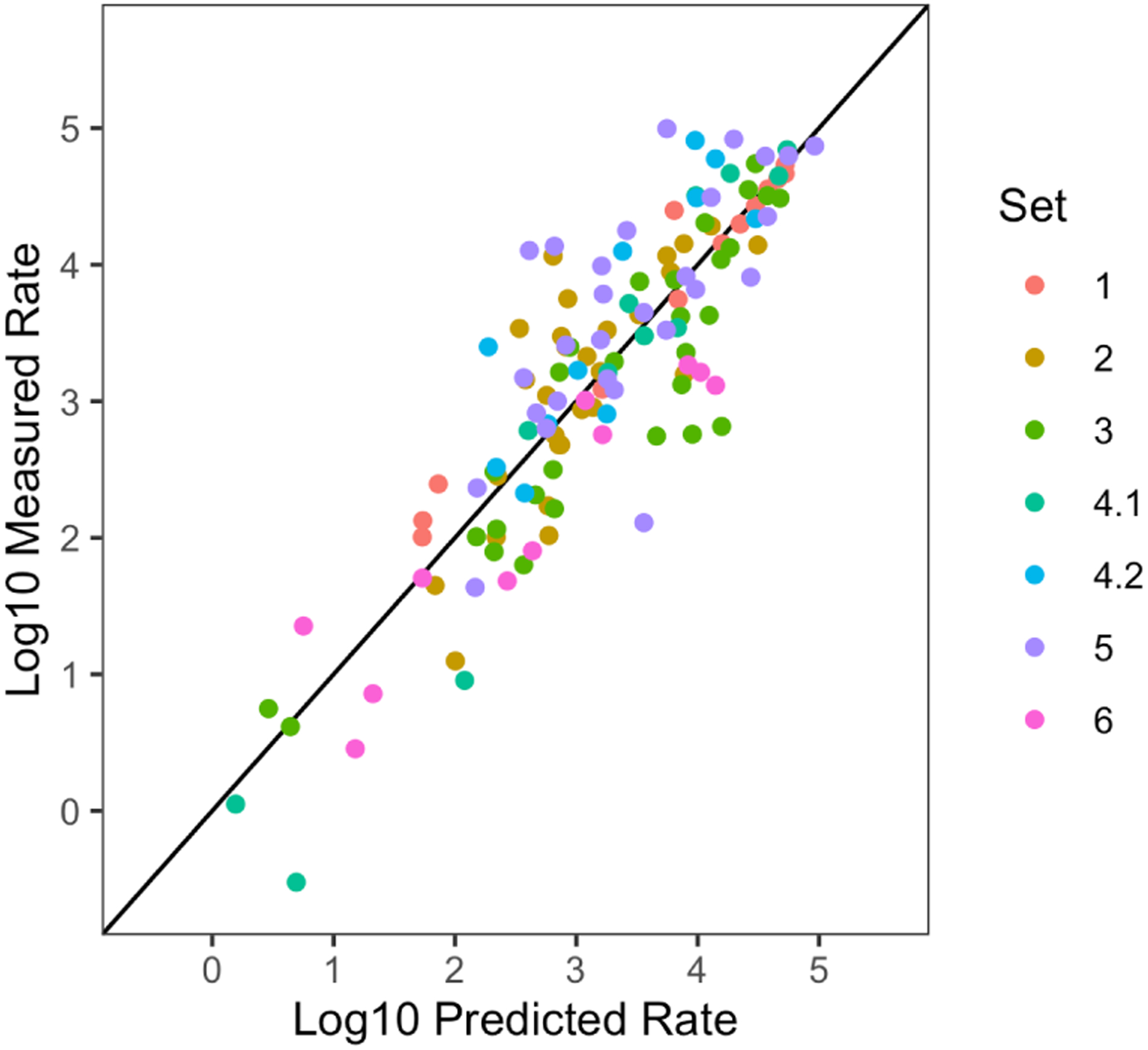
Comparison of experimentally measured translation initiation rates versus predictions made by OSTIR v1.0.0 using ViennaRNA version 2.4.18 for RNA energy calculations. Details of the experimental data, including a description of the different sets of sequences tested, are available in the original publication describing the RBS Calculator v1.0 (Salis et al., 2009).

Updating OSTIR to be compatible with ViennaRNA and newer RNA folding energy parameters required refitting coefficients in the underlying thermodynamic model (Reis & Salis, 2020; Salis et al., 2009; Salis, 2011). After making these changes, we verified that OSTIR has similar accuracy to the original RBS Calculator v1.0 (Figure 1). OSTIR predicts translation initiation rates for 53% of the test sequences within 2-fold of the experimentally measured values and for 91% of these sequences the predictions are within 10-fold of the measured values. Training data and R code for this statistical procedure are provided for users who want to work on further improving the model. In summary, we expect that OSTIR will be useful to researchers who want to model and engineer bacterial gene expression and incorporate these predictions into other software packages and computational pipelines.
